# Metabolic engineering of *Streptomyces explomaris* for increased production of the reverse antibiotic nybomycin

**DOI:** 10.1186/s12934-025-02860-4

**Published:** 2025-10-30

**Authors:** Wei Shu, Julian Stegmüller, Marta Rodriguez-Estevez, Christian Rückert-Reed, Jörn Kalinowski, Oleksandr Gromyko, Yuriy Rebets, Andriy Luzhetskyy, Christoph Wittmann

**Affiliations:** 1 Institute of Systems Biotechnology, Saarland University, Saarbrücken, Germany; 2 Pharmaceutical Biotechnology, Saarland University, Saarbrücken, Germany; 3 CEBITEC, Bielefeld University, Bielefeld, Germany; 4 Department of Genetics and Biotechnology, Ivan Franko National University of Lviv, Lviv, Ukraine; 5 Microbial Culture Collection of Antibiotic Producers, Ivan Franko National University of Lviv, Lviv, Ukraine; 6 Explogen LLC, Lviv, Ukraine

**Keywords:** *Streptomyces explomaris*, Metabolic engineering, Regulatory engineering, Nybomycin, Reverse antibiotic, Seaweed valorization

## Abstract

**Background:**

Nybomycin is a reverse antibiotic with selective activity against fluoroquinolone-resistant Gram-positive bacteria, including *Staphylococcus aureus*, making it a promising candidate to fight against antimicrobial resistance. However, its clinical development has been limited by the low production yields of native producers. To address this, we explored the heterologous expression of the nybomycin biosynthetic gene cluster (*nyb*) from the natural producer *S. albus subsp. chlorinus* NRRL B-24,108 in different marine and terrestrial *Streptomyces* hosts, aiming to boost production through targeted regulatory and metabolic engineering. We also evaluated the use of seaweed-derived hydrolysates as sustainable fermentation substrates.

**Results:**

Among several tested hosts, *S. explomaris* carrying the *nyb* gene cluster, produced the highest nybomycin titers. Global transcriptomic analysis identified transcriptional repression and precursor limitation as key bottlenecks. Deletion of the repressors *nybW* and *nybX* (NYB-1) significantly increased production, while further overexpression of genes boosting precursor supply (*zwf2*, *nybF*) led to the creation of NYB-3B, which reached a titer of 57 mg L^− 1^—fivefold higher than the previous benchmark. When cultivated on hydrolysates from commercial brown seaweed (*Himanthalia elongata*) without nutrient supplementation, NYB-3B achieved a titer of 14.8 mg L^− 1^.

**Conclusions:**

This study demonstrates the potential of *S. explomaris* as a chassis for high-level heterologous nybomycin production and its compatibility with renewable marine feedstocks. Regulatory and metabolic engineering effectively relieved key bottlenecks and improved precursor supply. The use of seaweed hydrolysates supports the development of sustainable nybomycin production. Collectively, these findings provide a valuable foundation for future efforts toward improved supply and clinical development of nybomycin.

**Supplementary Information:**

The online version contains supplementary material available at 10.1186/s12934-025-02860-4.

## Background

Nybomycin is a heterocyclic antibiotic with a unique structure distinct from common antibiotics like penicillins or tetracyclines. The compound exhibits activity against fluoroquinolone-resistant Gram-positive bacteria, including *Staphylococcus aureus* and *Enterococcus faecalis*, globally significant pathogens [1]. Nybomycin targets mutant *gyrA* (type II topoisomerase) containing an S84L substitution, counteracting acquired quinolone resistance and restoring susceptibility to DNA gyrase inhibitors [2]. Due to its ability to counteract quinolone resistance, it is classified as a reverse antibiotic (RA). In the context of escalating antimicrobial resistance (AMR), nybomycin represents a promising therapeutic candidate.

The clinical development of nybomycin is limited by its low production levels, which typically remain below 2 mg L^− 1^ in native *Streptomyces* strains [3, 4]. The biosynthetic pathway in *S. albus subsp. chlorinus* NRRL B-24,108 is encoded by a tightly regulated 35 kb biosynthetic gene cluster (BGC) [3]. The aromatic core structure of nybomycin is a fused tricyclic ring system, derived from polyketide precursors through enzyme-mediated cyclization and tailoring steps. Nybomycin synthesis depends on a complex metabolic network integrating inputs from the Embden–Meyerhof–Parnas (EMP), pentose phosphate (PP), and shikimate pathways. Key precursors include erythrose 4-phosphate (E4P), phosphoenolpyruvate (PEP), acetoacetyl-CoA (derived from malonyl-CoA), and NADPH as reducing equivalent [5]. Following construction of the aromatic core, the pathway proceeds through a series of post-polyketide tailoring reactions that yield the biologically active final compound. To date, heterologous expression systems have been explored as an alternative to low-yielding native producers of nybomycin. However, despite extensive pathway engineering [5] and the use of high-performance expression hosts [6], these efforts have achieved only modest improvement, with production titers remaining at 12 mg L^− 1^ [5]. This highlights the need to identify more suitable host strains capable of providing higher levels of nybomycin.

To identify an optimal heterologous host for nybomycin production, we evaluated a panel of *Streptomyces* strains for their ability to express the *nyb* biosynthetic gene cluster from the *S. albus subsp. chlorinus* NRRL B-24,108 [3]. Among the tested candidates, the marine isolate *S. explomaris* Je 1–4 achieved the highest nybomycin titer. Towards increased production, transcriptomic analysis of *S. explomaris* Je 1–4 identified key bottlenecks in nybomycin biosynthesis. These insights guided a combinatorial metabolic engineering approach. Among a family of engineered strains, *S. explomaris* NYB-3B exhibited the highest nybomycin titer, reaching 57 mg L^− 1^, representing a fivefold increase compared to previously reported values. To assess its applicability in low-cost bioprocesses, NYB-3B was further evaluated in complex media derived from minimally processed seaweed hydrolysates. Collectively, these results establish *S. explomaris* as a promising chassis for efficient and scalable heterologous nybomycin biosynthesis.

## Results and discussion

### Identification of *S. explomaris* as a high-performance host for nybomycin biosynthesis

**Fig. 1:**
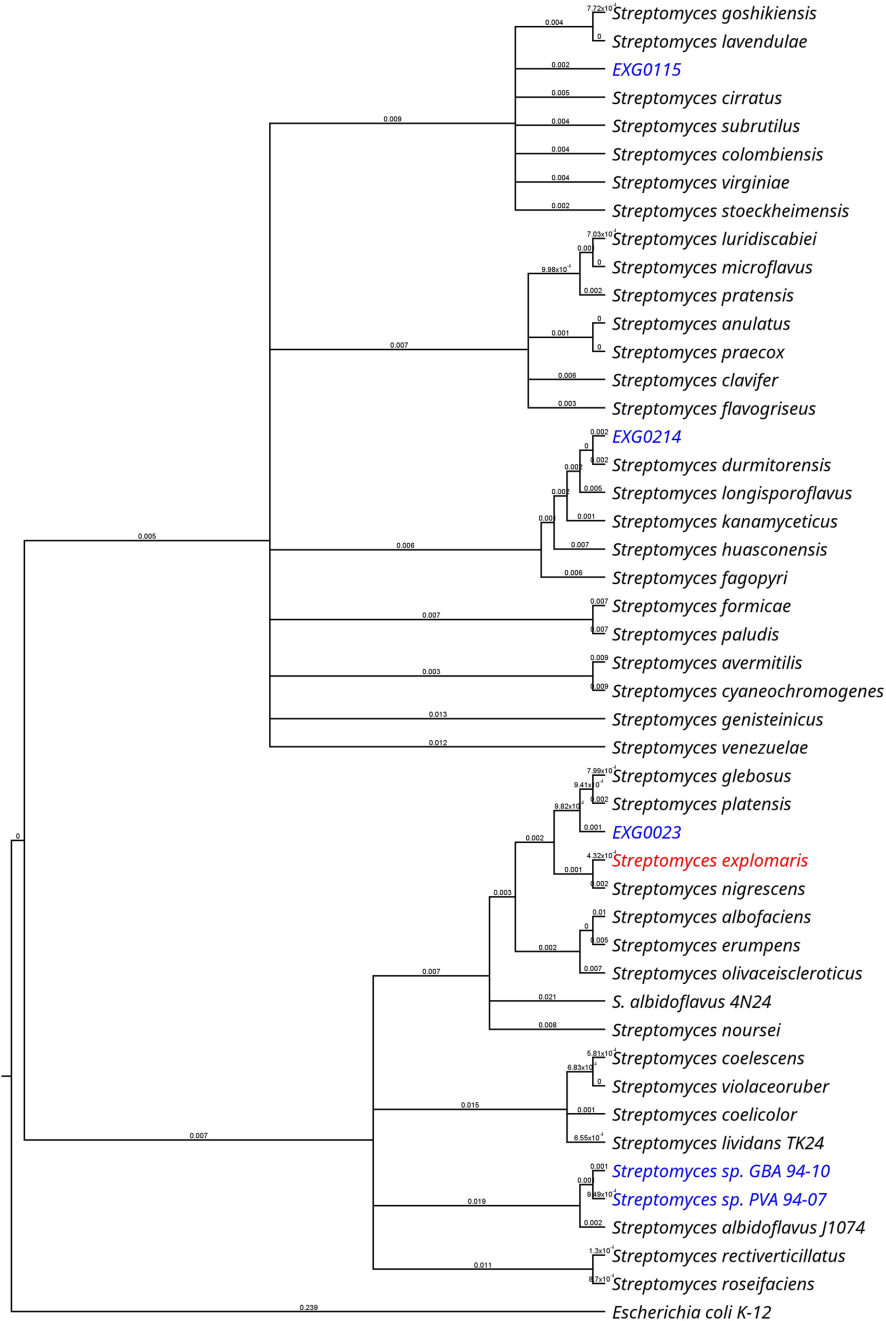
16 S rRNA gene-based phylogenetic analysis of *Streptomyces* species used as hosts for *nyb* gene cluster expression. The *E. coli* 16 S rRNA gene sequence was used as an outgroup. The typical heterologous *Streptomyces* hosts are shown in bold

**Fig. 2:**
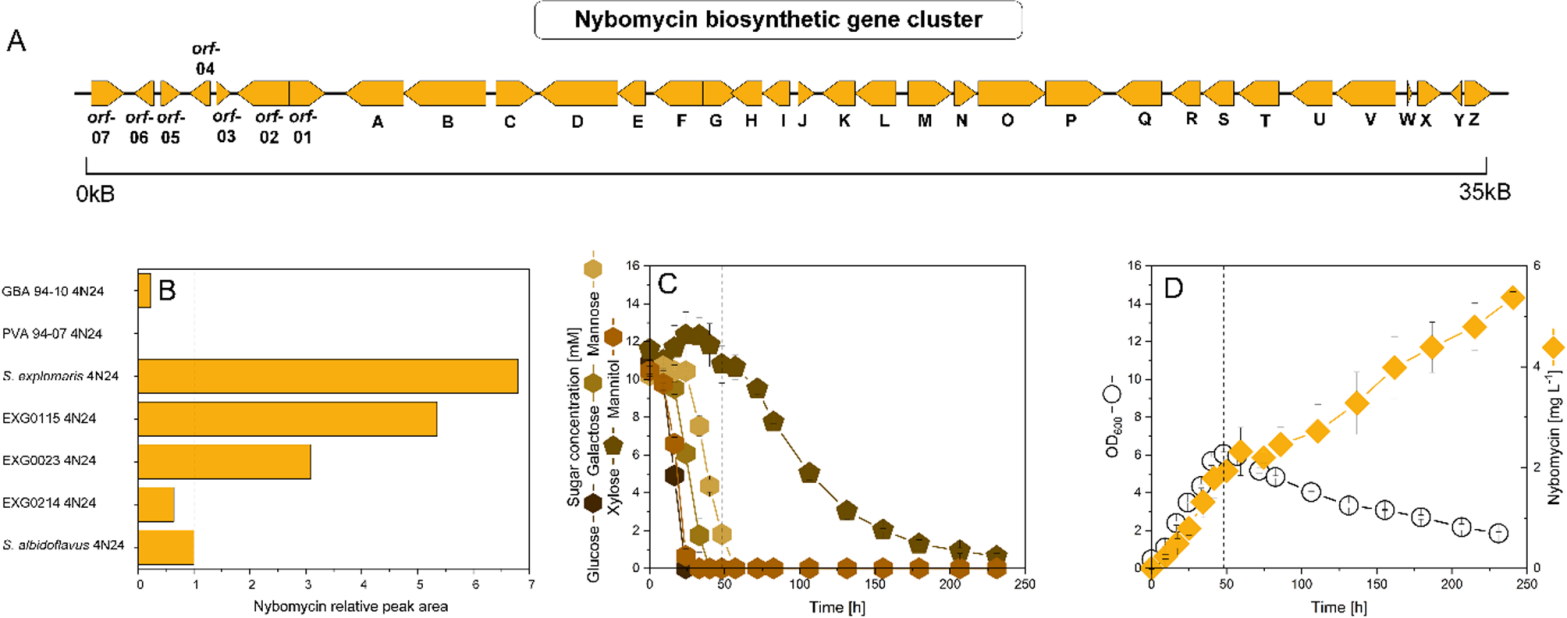
Evaluation of seven *Streptomyces* strains for heterologous nybomycin production. (A) Nybomycin biosynthetic gene (*nyb*) cluster. (B) Screening of seven terrestrial and marine *Streptomyces* isolates expressing the *nyb* cluster from BAC 4N24: the marine sponge-associated isolates *Streptomyces* sp. PVA 94 − 07 and *Streptomyces* sp. GBA 94 − 10, the marine rhizosphere-associated strain *S. explomaris*, the terrestrial strain *S. albidoflavus*, and three soil isolates EXG-0023, EXG-0115, and EXG-0214. Results in panel B represent single replicate screening experiments performed to identify promising hosts for further development. (C, D) Substrate consumption (C), growth, and nybomycin production (D) by *S. explomaris* 4N24. The strain was cultured in mineral medium containing a synthetic five-sugar mixture (mannitol, glucose, galactose, mannose, xylose) as the sole carbon source. Results in panels C and D represent biological triplicates (*n* = 3; mean ± SD)

Considering that species- and strain-level differences can lead to substantial variation in secondary metabolite biosynthesis, we evaluated a panel of so far unexplored *Streptomyces* strains to identify a suitable host for nybomycin production [7]. We evaluated three marine species: *S. explomaris* Je 1–4 isolated from coastal soil [8] and the two sponge-associated isolates *Streptomyces* sp. PVA 94 − 07 and *Streptomyces* sp. GBA 94 − 10 [9]. In addition, three terrestrial isolates (EXG0023, EXG0115, and EXG0214) were included into the study, based on their phylogenetic divergence from the typical *Streptomyces* hosts (Fig. 1). The *nyb* cluster, encoding the nybomycin production pathway from *S. albus subsp. chlorinus* NRRL B-24,108 (Fig. 2A) was cloned as BAC 4N24 and introduced into the six hosts (further designated as 4N24).

The recombinant strains were tested for nybomycin production in DNPM medium, along with *S. albidoflavus* 4N24, the parental strain of the so far best-performing nybomycin producer, as a benchmark [5]. Nybomycin production was observed in six of the seven strains (Fig. 2B), indicating the broad compatibility and functional expression of the biosynthetic gene cluster across diverse *Streptomyces* hosts. The strains exhibited considerable variation in yield. The recombinant *S. explomaris* 4N24 appeared to be the most efficient producer. It outperformed *S. albidoflavus* 4N24 by nearly sevenfold. Two of the three terrestrial isolates, EXG0023 and EXG0115, were also found to be superior over *S. albidoflavus* 4N24 with the latter one reaching fivefold higher yield. At the same time, the marine strains PVA 94 − 07 and GBA 94 − 10 demonstrated low and no detectable nybomycin production, respectively.

Based on its superior performance, *S. explomaris* 4N24 was selected for further development. When tested in minimal media with nine individual sugars as the sole carbon source, the strain was able to utilize glucose, mannose, galactose, xylose, and mannitol but not galactose, rhamnose, fucose, and arabinose. Notably, nybomycin production was strongly influenced by the type of sugar provided (Additional file 1, Fig. S2). The highest nybomycin titer was achieved on mannitol (11.0 mg L^− 1^), followed by glucose (7.5 mg L^− 1^), mannose (4.7 mg L^− 1^), and galactose (2.8 mg L^− 1^). In contrast, xylose was metabolized more slowly and supported only limited production (1.8 mg L^− 1^). Notably, the nybomycin titer of *S. explomaris* 4N24 on mannitol medium was nearly fourteenfold higher than that of *S. albidoflavus* 4N24, cultivated under identical conditions with the same carbon source [5].

*S. explomaris* 4N24 was next evaluated on a sugar mixture representative of biomass-derived hydrolysates [10], to assess its performance under more realistic conditions. When cultured on a mixture of five metabolizable sugars (2 g L^− 1^ each), *S. explomaris* 4N24 entered exponential growth immediately upon inoculation and demonstrated sequential sugar utilization (Fig. 2C). Nybomycin biosynthesis was favorably initiated early in the growth phase (Fig. 2D)—contrasting the typical late-onset production associated with nutrient limitation in most *Streptomyces* species [11], including nybomycin-producing *S. albidoflavus* 4N24 [5]. After growth ceased, nybomycin production paused for approximately 36 h but subsequently resumed, indicating a decoupling of production from active growth. Finally, *S. explomaris* 4N24 reached a nybomycin titer of 5.4 mg L^− 1^. With regard to substrate use, it co-consumed glucose, mannitol, and galactose during the first 24 h, followed by mannose utilization once glucose was depleted, enabling efficient biomass accumulation within 50 h. Xylose was slowly used up in later stages. These results demonstrated that the strain performs well on a biomass-representative sugar mixture, supporting both robust growth and effective product formation.

### Transcriptomic analysis reveals bottlenecks in precursor supply and cluster repression

**Fig. 3:**
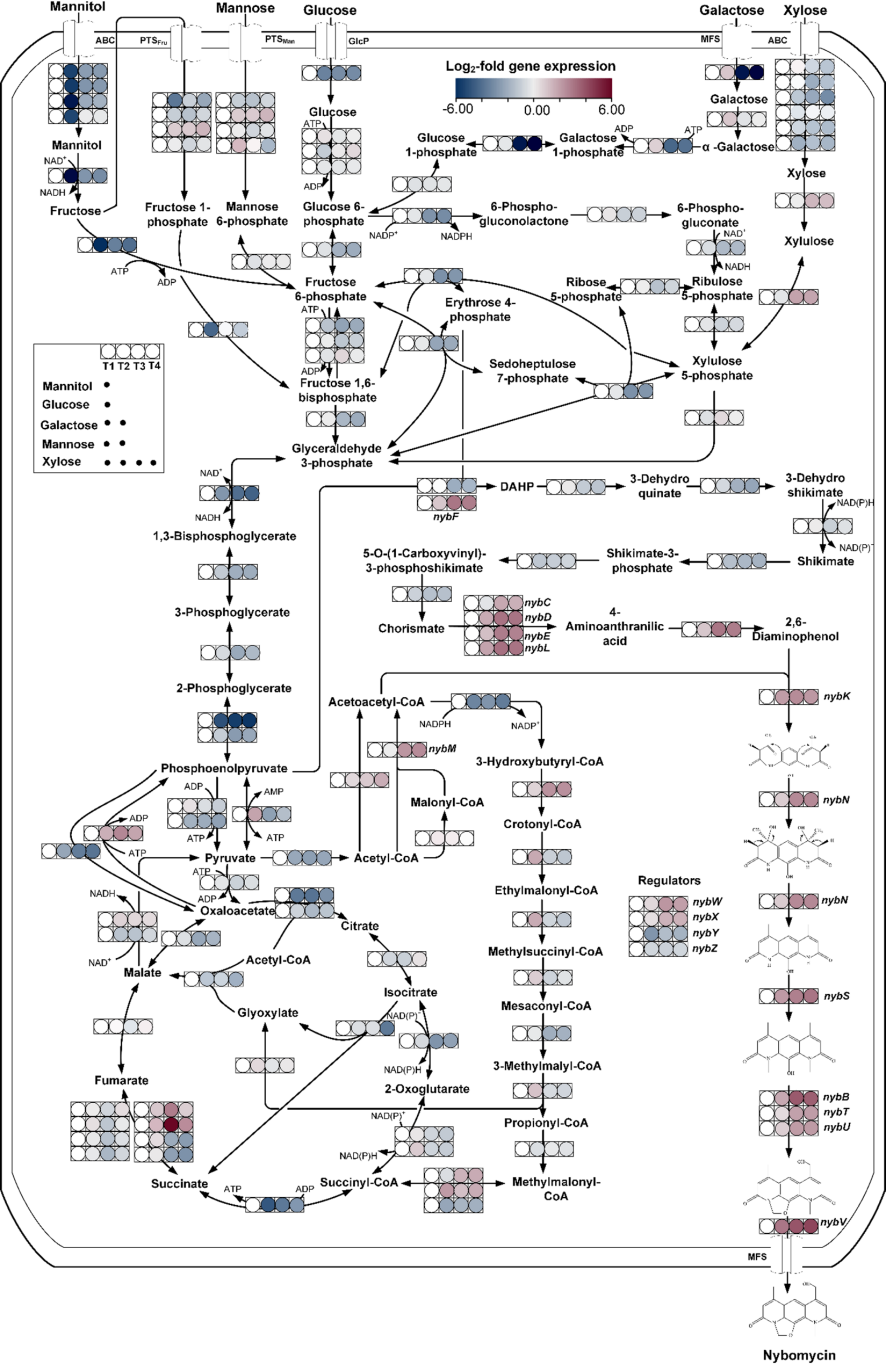
Time-resolved transcriptomic analysis of nybomycin-producing *S. explomaris* 4N24. The strain was cultured in a mineral freshwater medium containing a synthetic five-sugar mixture as sole source of carbon. RNA sequencing was conducted at four time points of the production process (Fig. 1), differing in substrate availability: T1 (17 h, used as reference), T2 (36 h), T3 (75 h), and T4 (175 h) (*n* = 3; mean ± SD)

The culture profile of *S. explomaris* 4N24 revealed complex dynamics; however, it did not provide sufficient resolution to identify specific cellular bottlenecks limiting productivity (Fig. 2). To gain deeper insights into the underlying regulatory and metabolic networks, we performed time-resolved RNA sequencing during the production process. Transcriptomic data collected at four time points (17 h, 36 h, 75 h, 175 h) revealed major transcriptional changes during the fermentation (Fig. 3). Using the initial time point (17 h) as a reference, a total of 3,193 genes—representing approximately 41.2% of the genome—were differentially expressed at 36 h, shortly after the depletion of glucose and mannitol (1,006 upregulated, 2,187 downregulated; adjusted *p* ≤ 0.05, fold change ≥ 1). The number of differentially expressed genes increased to 3,920 (50.6%) at 75 h, corresponding to the phase of resumed nybomycin production, and remained high at 175 h, with 3,835 genes (49.5%) showing significant expression changes.

Several transcriptional changes were observed in metabolic pathways that supply precursors for nybomycin biosynthesis (Fig. 3). Notably, the PP pathway, which provides E4P and NADPH, essential for building the aromatic nybomycin core, showed widespread downregulation during the stationary phase. Key genes affected included *zwf2* and *005996* (encoding glucose-6-phosphate dehydrogenase), 005997 (encoding transaldolase), as well as *tkt* and *005998* (encoding transketolase), suggesting a reduced flux through this pathway at later time points. On the other hand, PEP carboxykinase (*pepCK*,* 002843*) was highly expressed throughout the whole process, suggesting a sufficient supply of PEP, another essential precursor [3]. The shikimate pathway itself exhibited significant downregulation during the later stages. The DAHP synthase gene *005807*, which encodes the first committed enzyme of the pathway, showed a log_2_ fold change (FC) of − 2.37, indicating reduced flux into aromatic precursor biosynthesis. The nybomycin precursor acetoacetyl-CoA appeared to be readily available. The gene *004871*, encoding an enzyme that forms acetoacetyl-CoA, was upregulated (Fig. 3).

With regard to the *nyb* cluster, transcriptional analysis revealed pronounced changes in expression during the growth. Most genes encoding catalytic enzymes of the pathway were found upregulated, following carbon source depletion. The upregulation of the cluster-associated DAHP synthase gene *nybF*, encoding an alternative isoenzyme, suggested a possible pathway-specific redirection of flux toward nybomycin biosynthesis. Additionally, genes involved in the synthesis and incorporation of acetoacetyl-CoA, including *nybM* and *nybK*, were also upregulated, further supporting activation of specialized precursor supply routes.

The nybomycin biosynthetic pathway is controlled by four pathway-specific regulatory genes—*nybW*, *nybX*, *nybY*, and *nybZ* [3]. These regulators exhibited divergent transcription patterns (Fig. 3). *NybW*, a TetR-family repressor and putative repressor of the *nybQRSTUV* operon, was significantly upregulated. Similarly, the transcriptional regulator *nybX* was upregulated. Its homolog, *ScbR2* in *Streptomyces coelicolor*, functions as a repressor of actinorhodin biosynthesis and morphological differentiation [12]. This suggests that *nybX* may exert a similar repressive effect in *S. explomaris* 4N24. In contrast, both *nybZ* and *nybY* were strongly downregulated during the cultivation. These genes do not show significant homology to known activators or repressors, and their specific functions remain uncharacterized. Together, these findings suggested that combinatorial manipulation of precursor-supply routes and nybomycin pathway-specific regulators could represent a promising strategy for enhancing nybomycin production.

It was interesting to notice that the transcriptional responses of nybomycin-producing *S. explomaris* 4N24 (Fig. 3) and *S. albidoflavus* 4N24 [5] revealed marked differences in central metabolism and regulatory control. While *S. explomaris* showed strong downregulation of PP and shikimate pathway genes during the stationary phase, these remained relatively stable in *S. albidoflavus*. Similarly, the consistent upregulation of *nybW* and *nybX* in *S. explomaris* contrasted with their transient or modest expression in *S. albidoflavus*, indicating host-specific regulatory dynamics affecting cluster repression. These differences highlight how heterologous expression is shaped by the physiological and regulatory context of the host strain. Despite this, both strains upregulated *nybF* and PEP carboxykinase, suggesting a shared strategy to redirect carbon flux toward secondary metabolism via the shikimate pathway. This pathway serves as a crucial bridge between primary and secondary metabolism, supplying chorismate, a key precursor for various antibiotics and pigments [13].

### Regulatory engineering of the *Nyb* gene cluster

**Fig. 4:**
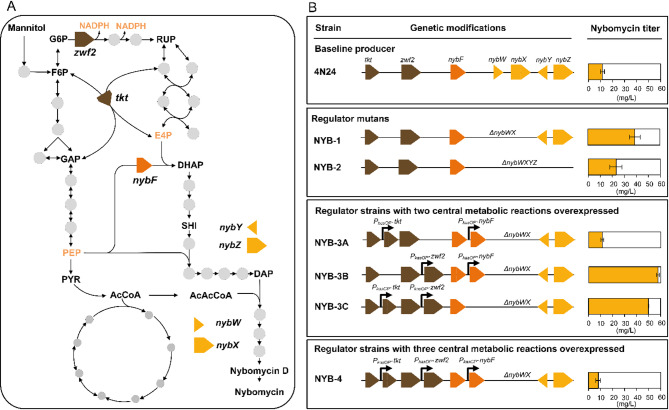
Metabolic pathway design for optimized nybomycin production in *S. explomaris*. **A** Metabolic engineering targets in primary and secondary metabolism. Creation of five generations of engineered nybomycin-producing strains through combinatorial modification of three complementary genetic modules encoding reactions of the pentose phosphate (PP), the shikimate, and the nybomycin biosynthetic pathways. **B** Nybomycin titers obtained in batch cultures on a mineral freshwater medium containing mannitol as sole source of carbon (*n* = 3; mean ± SD)

**Fig. 5:**
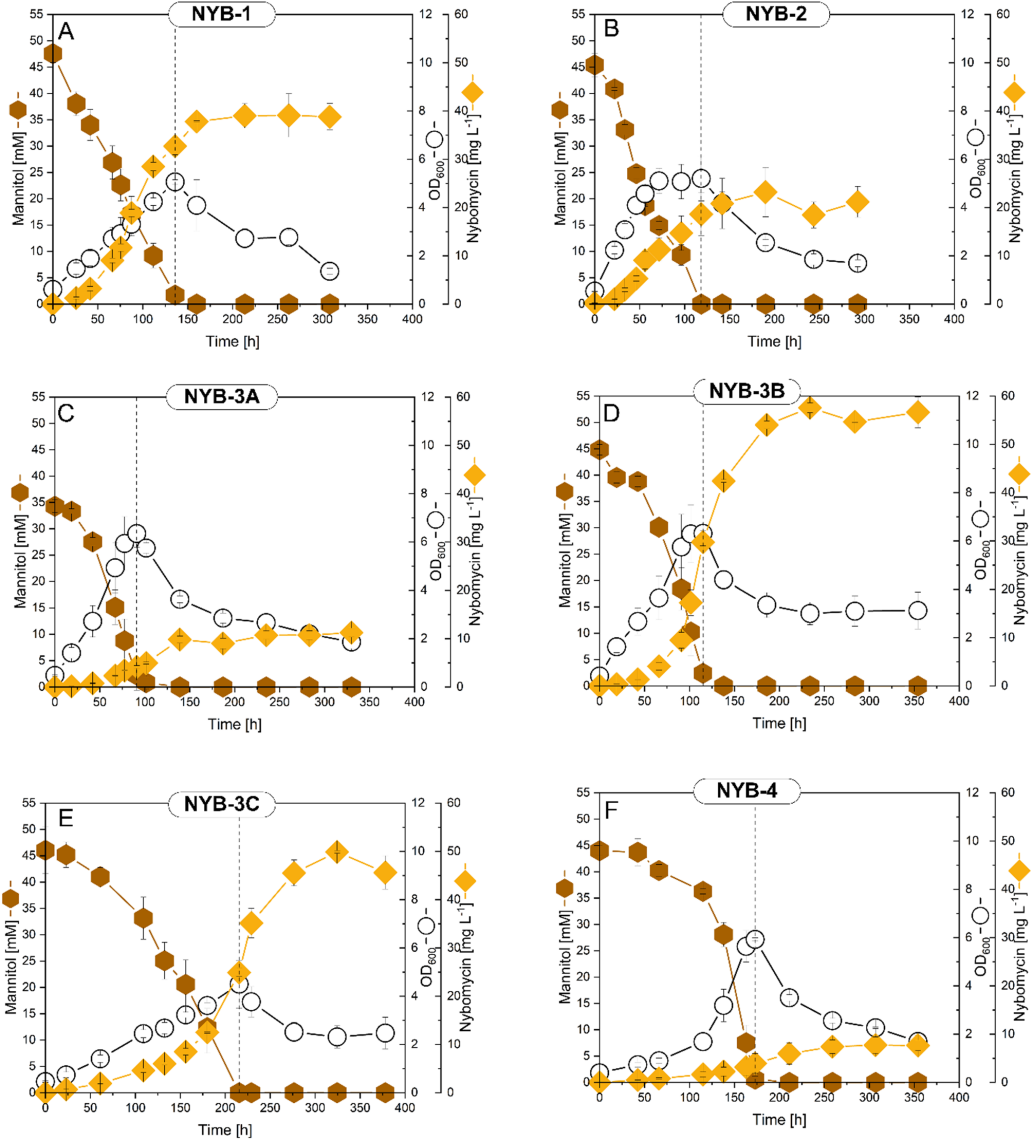
Nybomycin production in engineered *S. explomaris* strains. Cultivation profiles of strains NYB-1 (**A**), NYB-2 (**B**), NYB-3A (**C**), NYB-3B (**D**), NYB-3C (**E**), and NYB-4 (**F**) using mannitol-based minimal medium (*n* = 3; mean ± SD)

As shown, the *nyb* repressors *nybW* and *nybX* were upregulated in later growth stages, displaying a potential bottleneck for production (Fig. 3). Towards increased BGC expression, we therefore deleted *nybW* and *nybX* in the BAC 4N24 and introduced corresponding construct into *S. explomaris* (Fig. 4). The resulting strain *S. explomaris* NYB-1 (4N24 Δ*nybWX*) accumulated 39.1 mg L^− 1^ nybomycin in minimal mannitol medium (Figs. 4B and 5A), a 3.4-fold increase over the parental strain 4N24. These findings revealed that *nybWX* indeed played a dominant role in repressing the *nyb* biosynthetic cluster and that deleting these genes led to increased cluster expression.

In an alternative strategy, all four *nyb* regulators were deleted in the BAC 4N24 yielding *S. explomaris* NYB-2 (4N24 Δ*nybWXYZ*). The NYB-2 strain produced 23.2 mg L^− 1^ nybomycin, twice as high as its parental strain but significantly less than *S. explomaris* NYB-1 (Figs. 4B and 5B). Although the specific functions of *nybY* and *nybZ* remain unclear, the reduced nybomycin production observed in NYB-2 compared to NYB-1 suggested that *nybY* and/or *nybZ* may contribute positively to *nyb* BGC expression. Their removal appeared to partially counteract the beneficial effect of *nybW* and *nybX* deletion, indicating that *nybY* and *nybZ* are unlikely to function as repressors and may instead play a supportive or activating role in nybomycin biosynthesis.

In this study, nybomycin quantification was carried out from the total culture broth (cells plus supernatant), not from the supernatant fraction alone. Control extractions confirmed that two sequential rounds recovered approximately 97% of the total product, with approximately 70% present in the supernatant and 30% associated with the biomass. These results indicate that a significant proportion of nybomycin is secreted, but intracellular accumulation also contributes to the overall titer. The nybomycin biosynthetic cluster includes *nybV*, which encodes a putative efflux transporter [5]. In our engineered strains, regulatory deletions (Δ*nybWX*) led to a moderate increase in *nybV* expression (Fig. 5A). Importantly, previous work demonstrated that strong overexpression of *nybV* impaired growth and reduced production [5]. Together, these observations indicated that nybomycin export was sufficiently active in the optimized strains, while stronger overexpression did not promise further improvement.

### Metabolic engineering for increased precursor supply

Next, we aimed to increase flux through supporting pathways, i.e. the PP and shikimate pathways (Fig. 4A) to address potential limitations in precursor and NADPH supply, as indicated by the transcriptomic data (Fig. 3). The chosen combinatorial strategy was informed by previous systems metabolic engineering efforts, which had broadly demonstrated that the combinatorial implementation of complementary genetic targets is essential for developing high-efficiency production strains [14–21].

We employed a modular approach that combined enhancements to primary metabolism with regulatory pathway modifications. To this end, four distinct genetic modules comprising metabolic pathway genes were designed for genomic integration into the best-performing strain *S. explomaris* NYB-1. These created modules contained additional copies of genes encoding: (i) glucose-6-phosphate dehydrogenase (*zwf2*) to boost NADPH supply, (ii) transketolase (*tkt*) to increase E4P availability, and (iii) DAHP synthase (*nybF*) to enhance flux into the shikimate pathway (Fig. 4A). The polycistronic modules incorporated the target genes in different double combinations and one triple combination. Each module was placed under the control of the strong constitutive *P*_*kasOP*_ promoter [22] to ensure consistent expression throughout the process.

The four modules were individually integrated into strain NYB-1, resulting in three double-combination strains (NYB-3A, NYB-3B, NYB-3C) and one triple-combination strain (NYB-4). These engineered strains were cultivated in mannitol medium to evaluate their production performance. Notably, strains NYB-3B (57.6 mg L^− 1^) (Figs. 4B and 5D) and NYB-3C (49.9 mg L^− 1^) (Figs. 4B and 5E) showed a substantial increase in nybomycin titer—up to 47% higher than NYB-1. Both carried an additional copy of *zwf2*, underscoring its central role in enhancing nybomycin biosynthesis. Glucose-6-phosphate dehydrogenase, encoded by *zwf2*, catalyzes the first step of the oxidative PP pathway and is considered a pacemaker enzyme for NADPH generation [22], a key factor influencing natural product biosynthesis [23]. Although NYB-3B and NYB-3C both produced nybomycin efficiently, they exhibited pronounced differences in growth behavior, substrate utilization, and nybomycin accumulation dynamics. NYB-3B, in particular, demonstrated significantly greater vitality—it grew nearly twice as fast as NYB-3C, reached a 45% higher cell density, and attained peak nybomycin titers almost 100 h earlier. These data revealed that the genetic modifications not only boosted nybomycin biosynthesis but also markedly influenced central carbon metabolism and cellular physiology. The engineered gene combinations likely restructured intracellular fluxes, which collectively contributed to the observed differences in growth dynamics and production performance.

In contrast, NYB-3A maintained growth similar to the parental strain but failed to accumulate higher levels of nybomycin, especially during the stationary phase (Fig. 5C). The triple-combination strain NYB-4 (Fig. 5F) showed markedly reduced performance, with nybomycin titers even falling below those of the parental strain (Fig. 4B**)**. Its vitality was noticeably impaired: NYB-4 exhibited slower growth and reached a significantly lower cell density compared to NYB-3B, although the impairment was less severe than that observed in NYB-3C. These findings indicate that simultaneous overexpression of the three genes *zwf2*, *tkt*, and *nybF* led to substantial metabolic imbalances. A similar phenomenon has been observed in *S. coelicolor*, where the simultaneous overexpression of multiple genes disrupted, rather than enhanced, oviedomycin biosynthesis [23]. Collectively, our data suggest that the introduced genetic constructs affected both central carbon metabolism and nybomycin biosynthesis in a coordinated manner. This underscores the necessity of striking an optimal balance between growth and secondary metabolite production—evidently achieved in NYB-3B.

### Towards sustainable nybomycin production using seaweed hydrolysates

**Fig. 6:**
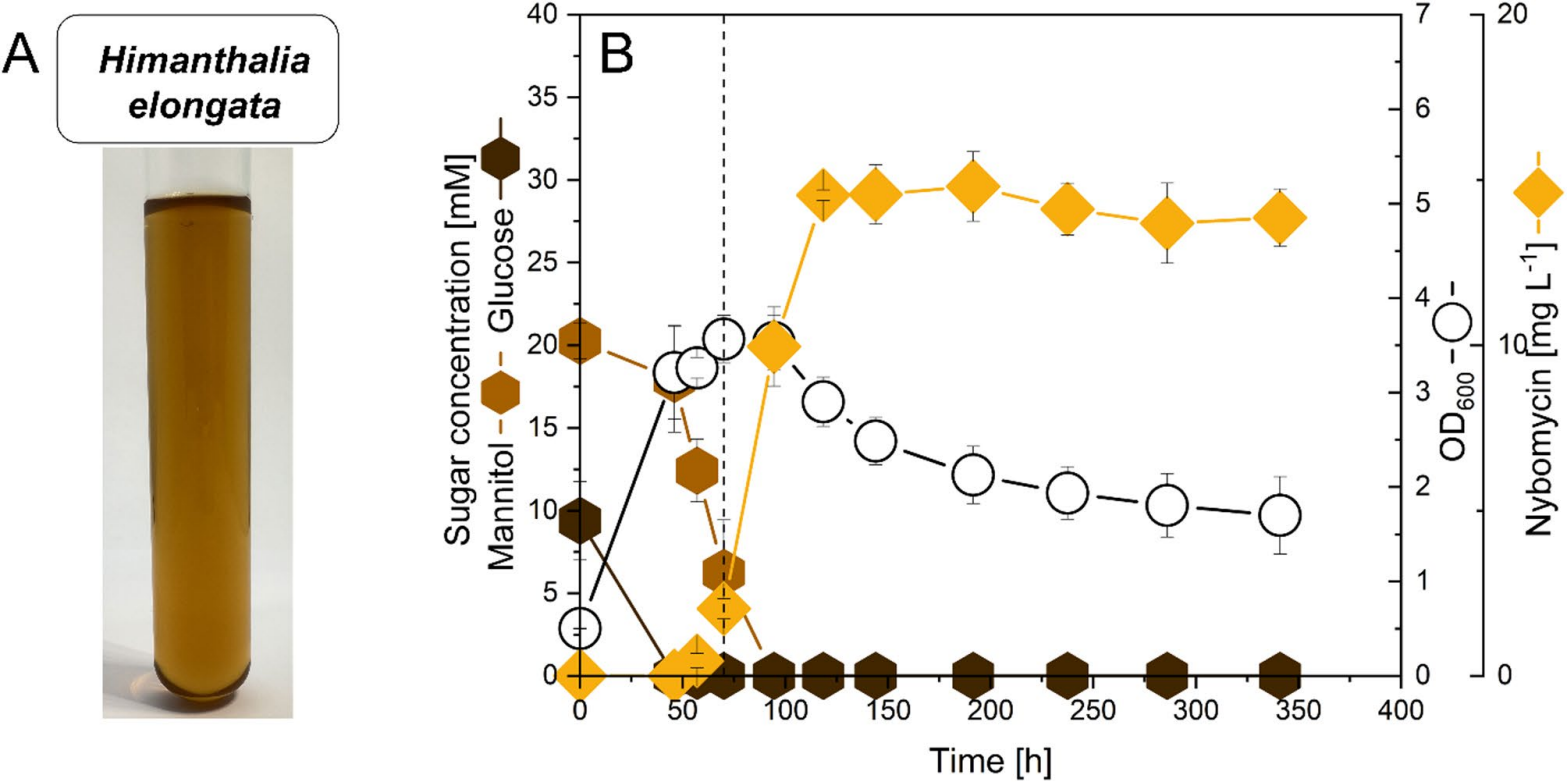
Valorization of brown seaweed for nybomycin production using the engineered producer *S. explomaris* NYB-3B. **A** Raw hydrolysate, used as fermentation substrate. **B** Growth, substrate consumption, and nybomycin production of NYB-3B in hydrolysate medium (90% extract, 10% buffer, no additional supplementation). The experiment was conducted for 350 h, and the complete cultivation profile is shown. Results represent biological triplicates (*n* = 3; mean ± SD)

Given the wide substrate spectrum of *S. explomaris*, we decided to explore the feasibility of using seaweed-derived raw materials for nybomycin biosynthesis. As shown, *S. explomaris* metabolized glucose, mannose, galactose, xylose, and mannitol, which are commonly found in marine seaweed and seaweed-based hydrolysates [24]. Such feedstocks offer a sustainable alternative to conventional carbon sources. Seaweed is fast-growing, non-competitive with food crops, and requires neither arable land nor freshwater [25] and has been shown as suitable fermentation substrate for *Streptomyces* strains [26]. First, we investigated the potential to valorize *Himanthalia elongata*, a commercial brown seaweed (Fig. 6). Hot water extraction of the dried, ground material, followed by enzymatic hydrolysis and filtration, yielded a hydrolysate that contained mannitol and glucose (Fig. 6A). A fermentation medium was then prepared consisting of 90% hydrolysate and 10% buffer solution without further supplementation. *S. explomaris* NYB-3B sequentially utilized glucose and mannitol for growth and achieved a nybomycin titer of 14.8 mg L^− 1^ (Fig. 6B). The nybomycin titer was lower than that achieved on synthetic mannitol medium. This was partly attributable to the reduced initial total sugar concentration (30 mM versus 45 mM), of which 30% was glucose—a carbon source shown to be less effective for nybomycin production. Interestingly, the strain did not accumulate any product during the growth phase, in contrast to its behavior on synthetic medium. This suggests that the salt-rich environment induced complex metabolic changes, as has been observed in other microbes [27]. Further research is needed to elucidate these underlying mechanisms and optimize *S. explomaris* for increased nybomycin production using seaweed-derived substrates. Nevertheless, these findings demonstrate the feasibility of using untreated seaweed hydrolysates as a sustainable carbon source to produce the reverse antibiotic.

While the engineered strain NYB-3B achieved the highest reported nybomycin titer to date, further improvements will likely require optimization of the production process itself. One important consideration is the potential toxicity of nybomycin toward its host. Our extraction analysis revealed that, in addition to the approximately 70% of nybomycin detected in the culture supernatant, a considerable fraction (about 30%) remained associated with the biomass. This distribution indicates that intracellular or cell-associated accumulation may contribute to cellular stress. In situ product removal strategies, such as the addition of macroporous resin, have been successfully applied in polyketide fermentations to alleviate product toxicity, improve yield, and simplify downstream recovery [28–30]. Beyond the present study, resin-assisted adsorption could represent a promising route to further enhance nybomycin productivity and process robustness. Such approaches may be evaluated in future studies.

## Conclusion

In this study, we successfully enhanced nybomycin production in the marine actinomycete *S. explomaris* through a combination of transcriptomics-driven regulatory and metabolic engineering, while also demonstrating the feasibility of using renewable marine feedstocks as sustainable fermentation substrates. Targeted deletion of pathway-specific repressors (*nybW* and *nybX*) and overexpression of key metabolic genes (*zwf2*, *tkt*, and *nybF*) significantly improved flux toward the shikimate pathway and alleviated transcriptional bottlenecks (Fig. 4B). The resulting strain, NYB-3B, achieved a nybomycin titer of 57 mg L^− 1^—nearly fivefold higher than the previous benchmark producer *S. albidoflavus* NYB-9 [5]. Importantly, *S. explomaris* 4N24 and its engineered derivatives efficiently utilized enzymatically processed brown seaweed hydrolysate, rich in glucose and mannitol, without the need for additional supplementation.

This work suggests that *S. explomaris* holds promise as a suitable host for polyketide biosynthesis and may support the development of more sustainable approaches to nybomycin production. While encouraging, further optimization will be required to improve process scalability, including refinement of fermentation conditions, management of variability in seaweed-derived feedstocks, and development of efficient downstream processing. These findings provide a foundation for future studies aimed at advancing nybomycin as a potential therapeutic option for combating drug-resistant pathogens.

## Materials and methods

### Strains and plasmids

*S. explomaris*, an isolate from the coastal rhizosphere soil of *Juniperus excelsa* M. Bieb (Crimean Peninsula) [8], the sponge-associated marine strains *Streptomyces* sp. PVA 94 − 07 and *Streptomyces* sp. GBA 94 − 10 [9], and *S. albidoflavus* 4N24 (*S. albus* 4N24) [5] were obtained from previous work. *Streptomyces* sp. EXG0023 and EXG0115 were isolated from the rhizosphere of *Echeveria agavoides* and EXG0214 – from the rhizosphere of *Citrus limon* (Explogen LLC, unpublished data). *Escherichia coli* DH10B (Thermo Fisher Scientific, Karlsruhe, Germany) was used for cloning, while *E. coli* ET12567/pUZ8002 served as the donor strain for intergeneric conjugation [31]. BAC 4N24 containing the *nyb* BGC was isolated from *S. albus subsp. chlorinus* [3]. A list of all strains and plasmids used and created in this study is provided Table 1.

**Table 1:** Strains and plasmids

	Description	References
*Escherichia coli*		
DH10B	F– mcrA Δ(mrr-hsdRMS-mcrBC) φ80lacZΔM15 ΔlacX74 recA1 endA1 araD139 Δ (ara-leu)7697 galU galK λ– rpsL (StrR) nupG	Thermo Fisher Scientific
ET12567/pUZ8002	Methylation deficient ET12567 containing nontransmissible RP4 derivative plasmid pUZ8002, Cm^R^, Kan^R^	[31]
*Streptomyces*		
* Streptomyces* sp.		
PVA 94 − 07	Natural isolate	[9]
PVA 94 − 07 4N24	Expression of BAC 4N24	This work
GBA 94 − 10	Natural isolate	[9]
GBA 94 − 10 4N24	Expression of BAC 4N24	This work
EXG0023	Natural isolate	This work
EXG0023 4N24	Expression of BAC 4N24	This work
EXG0115	Natural isolate	This work
EXG0115 4N24	Expression of BAC 4N24	This work
EXG0214	Natural isolate	This work
EXG0214 4N24	Expression of BAC 4N24	This work
* S. albidoflavus*		
4N24	Expression of BAC 4N24	[3]
* S. explomaris*		
DSM 117,375^T^	Natural isolate	[8]
4N24	Expression of BAC 4N24	This work
NYB-1	4N24, in-frame deletion of *nybWX*	This work
NYB-2	4N24, in-frame deletion of *nybWXYZ*	This work
NYB-3A	NYB-1, integration of *nybF* (FM076 RS29120) from *S. albus* subsp. *chlorinus* NRRL B- 24,108 and *tkt* (B591 RS24355) from *Streptomyces* sp. GBA 94–10 under control of *P*_kasOP*_	This work
NYB-3B	NYB-1, integration of *nybF* (FM076 RS29120) from *S. albus* subsp. *chlorinus* NRRL B- 24,108 and *zwf2* (B591 RS24345) from *Streptomyces* sp. GBA 94–10 under control of *P*_kasOP*_	This work
NYB-3C	NYB-1, integration of *tkt* (B591 RS24355) and *zwf2* (B591 RS24345) from *Streptomyces* sp. GBA 94–10 under control of *P*_kasOP*_	This work
NYB-4	NYB-1, integration of *nybF* (FM076 RS29120), *tkt* (B591 RS24355), and *zwf2* (B591 RS24345) under control of *P*_kasOP*_	This work
NYB-5A	NYB-2, integration of *nybF* (FM076 RS29120) and *tkt* (B591 RS24355) under control of *P*_kasOP*_	This work
NYB-5B	NYB-2, integration of *nybF* (FM076 RS29120) and *zwf2* from *Streptomyces* sp. GBA 94–10 under control of *P*_kasOP*_	This work
NYB-5C	NYB-2, integration of *tkt* (B591 RS24355) and *zwf2* (B591 RS24345) under control of *P*_kasOP*_	This work
NYB-6	NYB-2, integration of *nybF* (FM076 RS29120), *tkt* (B591 RS24355), and *zwf2* (B591 RS24345) under control of *P*_kasOP*_	This work
Plasmids/BAC		
* pBT1HP*	Integrative plasmid containing *oriT*, *attP*, *int* phiBT1, *hph*, PermE*, *tfd*	[5]
* pBT1H*	Integrative plasmid containing *oriT*, *attP*, *int* phiBT1, *hph*	[5]
4N24	BAC including the *nyb* gene cluster from *S. albus* subsp. *chlorinus* NRRL B- 24,108	[3]
4N24 *ΔnybWX*	4N24 with in-frame replacement of *nybW* (FM076 RS29200) and *nybX* (FM076 RS29205) by Kan^R^	This work
4N24 *ΔnybWXYZ*	4N24 with in-frame replacement of *nybW* (FM076 RS29200), *nybX* (FM076 RS29205), *nybY* (FM076 RS29210), and *nybZ* (FM076 RS29215) by Kan^R^	This work
*pBT1H P*_kasOP*_ *nybF tkt*	Integrative plasmid containing *P*_kasOP*_ *nybF*, *tkt*, Hyg^R^	This work
*pBT1H P*_kasOP*_ *nybFzwf2*	Integrative plasmid containing *P*_kasOP*_ *nybF zwf2*, Hyg^R^	This work
*pBT1H P*_kasOP*_ *tkt zwf2*	Integrative plasmid containing *P*_kasOP*_ *tkt zwf2*, Hyg^R^	This work
*pBT1H P*_kasOP*_ *nybF tkt zwf2*	Integrative plasmid, containing *P*_kasOP*_ *nybF tkt zwf2*, Hyg^R^	This work

### Genome sequencing and phylogenetic analysis

Genomic DNA was extracted using the NucleoSpin Microbial DNA Kit (Macherey-Nagel, Germany) according to the manufacturer’s protocol. Illumina shotgun libraries were prepared with the TruSeq DNA PCR-Free Kit and sequenced on the Illumina NovaSeq 6000 platform in 2 × 150 bp paired-end mode (Illumina, USA). Quality control of the raw reads was performed using FastQC [32]. High-quality reads (≥ 100× coverage) were assembled with Newbler v3.0 [33]. Genome annotation was carried out using the NCBI Prokaryotic Genome Annotation Pipeline (PGAP) v6.7 [34]. For phylogenetic analysis, 16 S rRNA gene sequences were aligned using the MUSCLE algorithm [35]. Evolutionary distances were calculated with the Jukes-Cantor model [36], and a phylogenetic tree was constructed using the Neighbor-Joining method [37]. The tree was visualized in Geneious Prime v2025.2.1 (Dotmatics, USA). The 16 S rRNA gene of *Escherichia coli* MG1655 (GenBank accession no. CP044355.1) was used as the outgroup.

### Genetic engineering

Cloning strategies were designed using SnapGene software (GSL Biotech LLC, San Diego, USA). DNA cloning, vector construction, and DNA transfer into *E. coli* DH10B, followed by conjugative transfer into *Streptomyces*, were performed as previously described [5].

The gene cluster for nybomycin biosynthesis was taken from BAC 4N24 from previous work [3]. Following plasmid isolation using the QIAprep Spin MiniPrep Kit (Qiagen, Hilden, Germany), the constructs were verified by PCR, restriction digestion, and sequencing. The selected plasmid was subsequently introduced into the methylation-deficient *E. coli* ET12567/pUZ8002 via electroporation. The resulting transformants served as donor strains for intergeneric conjugation, facilitating the transfer of the plasmid into *Streptomyces* strains [31].

For conjugation, *E. coli* ET12567/pUZ8002 was cultivated in 10 mL LB medium with appropriate antibiotics until an OD_600_ of 0.4–0.6 was reached. To remove antibiotics, the cells were harvested by centrifugation, washed twice with fresh LB medium, and resuspended in 0.5 mL LB medium. Recipient spores were collected from mannitol-soy flour (MS) agar plates following incubation at 30 °C for 5–7 days, resuspended in 1 mL deionized water, and used as the recipient. Donor and recipient cells were mixed at a 1:1 ratio and spread onto MS agar plates supplemented with 20 mM CaCl₂, followed by incubation at 30 °C for 16 h. After incubation, 1 mL of water containing 200 µg mL⁻¹ phosphomycin and 50 µg mL⁻¹ hygromycin B was overlaid on the plate, and incubation continued at 30 °C for 3–5 days until exconjugants emerged. Exconjugants were subsequently transferred to fresh MS plates containing phosphomycin and hygromycin for further selection. The correctness of the exconjugants was verified by PCR and sequencing. Integration of the recombinant DNA into the chromosome was mediated by site-specific recombination involving φBT1 integrase [31]. Different polycistronic modules were constructed to express *nybF*, isolated from BAC 4N24, along with *zwf2* (glucose 6-phosphate dehydrogenase) and *tkt* (transketolase) both derived from genomic DNA of *Streptomyces* sp. PVA 94 − 07. These genes were combined in different configurations and placed under the regulation of the *P*_*kasOP*_ promoter [38]. Deletion of target genes in BAC 4N24 was achieved using the *E. coli* Red/ET recombination system [39]. In brief, genes targeted for deletion were replaced by an antibiotic resistance cassette flanked by appropriate homology arms via Red/ET recombination. The resulting BAC construct was subsequently introduced into the selected *Streptomyces* host by conjugation. The desired genomic modifications were verified by PCR and sequencing [5].

### Extraction of sugars from macroalgae

Dried brown seaweed (*Himanthalia elongata*, PureRaw, Klötze, Germany) was pulverized (50 g), mixed with 500 mL of deionized water, and heated (121 °C, 15 min). Enzymatic hydrolysis of the suspended biomass was performed by adding a blend of Celluclast (1.5 L) and Viscozyme L (Sigma‒Aldrich, Steinheim, Germany) at a concentration of 0.01 g per gram of dry biomass. After incubation (48 h, 50 °C, pH 5.5), the hydrolysate was centrifuged (4,500 × g, 15 min, 4 °C) to remove solid debris, adjusted to pH 7.0 (6 M NaOH) and sterilized (121 °C, 15 min).

### Growth and production media

*E. coli* and *Streptomyces* strains were cultivated in Luria–Bertani (LB) broth (Becton & Dickinson, Heidelberg, Germany). For the solid media, LB broth was supplemented with 20 g L^− 1^ Difco agar (Becton & Dickinson). When needed, antibiotics were added at the following concentrations: hygromycin B at 50 µg mL^− 1^ for *Streptomyces* and 100 µg mL^− 1^ for *E. coli*, kanamycin at 50 µg mL^− 1^, phosphomycin at 200 µg mL^− 1^, and apramycin at 50 µg mL^− 1^. For sporulation, *Streptomyces* strains were cultivated on mannitol-soy flour (MS) agar, which contained 20 g mannitol (Sigma‒Aldrich), 20 g soy flour (Schoenenberger Hensel, Magstadt, Germany), and 20 g agar (Becton Dickinson) per liter. For liquid cultures, International Project (ISP) No. 1 medium [40] was used for the initial preculture, with the following composition per liter: 5 g of tryptone (Becton & Dickinson) and 3 g of yeast extract (Becton & Dickinson), with the pH adjusted to 7.0-7.2. For the screening of different *Streptomyces* hosts for nybomycin production, the main cultures were grown in liquid DNPM medium (40 g L^− 1^ dextrin, 7.5 g L^− 1^ soytone, 5 g L^− 1^ yeast extract, and 21 g L^− 1^ MOPS, pH 6.8). For further studies, the second preculture and main cultivation medium, both chemically defined, were prepared in two different setups. In the first setup, the medium was prepared with deionized water and contained the following per liter: 0.4 g of MgCl_2_·6H_2_O, 0.0265 g of CaCl_2_·2H_2_O, 7 g of (NH_4_)_2_SO_4_, 20 g of MOPS buffer (pH 7.0), 0.5 g of K_2_HPO_4_, and 40 mL of vitamin solution (10 mg of biotin, 4 mg of thiamine-HCl, 40 mg of inositol, 8 mg of pantothenic acid, and 4 mg of pyridoxine-HCl per liter). In the second setup, the deionized water was replaced with artificial seawater [41], which had the following composition per liter: 27.13 g NaCl, 1.17 g CaCl_2_, 3.38 g MgSO_4_, 2.5 g MgCl_2_, 0.74 g KCl, and 0.21 g NaHCO_3_. The rest of the medium composition remained unchanged. Different carbohydrates were added as the sole source of carbon to each setup either individually or in combination. When tested individually, the medium was supplemented with 10 g L^− 1^ of a single sugar, as described below. For growth on sugar mixtures, different combinations were prepared, each containing 2 g L^− 1^ of each included sugar. These conditions were applied separately to both the deionized water and artificial seawater setups to assess the influence of seawater components on metabolism. For cultivation in seaweed hydrolysates, ISP 1 medium was used for preculture, and the main cultivation medium consisted of 90% (v/v) macroalgal extract mixed with 10% (v/v) MOPS buffer (2 M, pH 7.0).

### Growth and nybomycin production experiments

For the initial host screening (Fig. 2B), nybomycin production was measured in single replicate cultures in DNPM medium. These experiments served to identify promising *Streptomyces* strains for further development and were not intended for quantitative comparison. All subsequent experiments, including regulatory and metabolic engineering (Figs. 4, 5 and 6), were conducted in biological triplicates, and results are presented as mean ± standard deviation (SD). Liquid cultivation in defined media was conducted in 500 mL baffled shake flasks with a working volume of 10%. To enhance mixing, 30 g of soda-lime glass beads (5 mm, Sigma‒Aldrich) were added [42]. The cultures were incubated on an orbital shaker (Multitron, Infors AG, Bottmingen, Switzerland) at 230 rpm, 75% relative humidity, and 28 °C. For standard cultivation, spores from a single colony were inoculated into ISP 1 medium for the first preculture and incubated overnight. The cells were then harvested by centrifugation (5000 ×*g*, 5 min, 25 °C) and resuspended in the second preculture medium. Following an additional overnight incubation, the cells were collected again under the same conditions and used to inoculate the main culture in defined medium. For cultivation in seaweed hydrolysate-based media, spores from a single colony were first inoculated into ISP 1 medium and incubated overnight. The cells were then harvested (5000 ×*g*, 5 min, 25 °C) and directly transferred into the main cultivation medium.

### Quantification of the cell concentration

Growth was monitored primarily by measuring the optical density at 600 nm (OD_600_). Additionally, the dry cell weight (CDW) was determined gravimetrically [43]. Systematic measurements for *S. explomaris* over entire culture periods established a linear correlation to infer CDW from OD_600_ readings: CDW (g L^− 1^) = 0.56 × OD_600_, so that OD_600_ readings could be considered representative of cell growth (Supplementary Information).

### Quantification of sugar and sugar alcohols

Glucose, mannose, fructose, rhamnose, galactose, fucose, xylose, arabinose, and mannitol were quantified using high-performance liquid chromatography (HPLC) (1260 Infinity Series, Agilent, Darmstadt, Germany) equipped with a Nucleogel Sugar Pb column (10 μm, 300 × 7.8 mm, Macherey-Nagel, Düren, Germany) and a Nucleodur C18 Isis guard column (3 μm, 100 × 3 mm, Macherey-Nagel). Demineralized water was used as the mobile phase at a flow rate of 0.4 mL min^− 1^ and a column temperature of 80 °C. Detection was performed via refractive index measurement, and quantification was achieved via the use of external calibration standards.

### Extraction and quantification of nybomycin

Extraction and quantification of nybomycin were performed as previously described [5]. For compound identification, nybomycin was extracted from the culture supernatant and analyzed by LC–MS to confirm fragmentation patterns and accurate masses. For quantitative analysis, however, the compound was extracted from the total culture broth (cells plus supernatant). In this approach, two sequential liquid–liquid extraction steps were performed using ethyl acetate, and the pooled extracts were freeze-dried and re-dissolved in a mixture of methanol and DMSO (1:1) prior to LC-ESI-MS/MS analysis. Control experiments confirmed that two rounds of extraction recovered approximately 97% of the total nybomycin. Distribution analysis showed that approximately 70% of the product was present in the supernatant, while 30% was associated with the biomass.

### Transcriptome analysis

RNA extraction and sequencing were performed according to previously established protocols [26]. Briefly, cells (1 mL culture broth) were harvested by centrifugation (16,000 x*g*, 4 °C, 1 min) at four different time points (17 h, 36 h, 75 h, 175 h) and immediately flash-frozen in liquid nitrogen. RNA was isolated using the Qiagen RNA Mini Kit (Qiagen, Hilden, Germany), following the manufacturer’s instructions. Residual DNA was removed with 10 U RNase-free DNase I (Thermo Scientific) for 1 h, supplemented with RiboLock RNase inhibitor (Thermo Scientific). The RNA underwent additional purification with the same kit. RNA quality was assessed using the Trinean Xpose (Trinean NV, Gentbrugge, Belgium) and Agilent RNA 6000 Nano Kit on an Agilent 2100 Bioanalyzer (Agilent Technologies, Böblingen, Germany). Ribosomal RNA was depleted using the Ribo-Zero rRNA Removal Kit (Illumina, San Diego, CA, USA), and depletion was confirmed with the Agilent RNA 6000 Pico Kit on an Agilent 2100 Bioanalyzer (Agilent Technologies). cDNA libraries were prepared using the TruSeq Stranded mRNA Library Prep Kit (Illumina) and sequenced on an Illumina HiSeq 1500 platform (2 × 75 bp paired-end reads). Read trimming/filtering was performed using trimmomatic v0.39 [44] in PE mode with trimmers SLIDINGWINDOW:4:15 and MINLEN:25. Reads were aligned to the S. explomaris_4N24 genome (CP110836.1) using Bowtie2 [45], with an adjusted maximum read pairing distance of 600 bases. ReadXplorer 2.2.3 [46] was used for read visualization and FeatureCounts [47] as used for count calculations with parameters -O, -M, -t gene, -g locus_tag, and -s 2. Differential gene expression (DGE) was analyzed with DESeq2 [48]. The data were validated for statistical significance using PCA. The obtained raw sequencing reads and processed datasets (input matrix and normalized read counts) are publicly available at GEO (*GSE291039*). A Student’s t-test was applied to identify genes with a log₂ fold change (FC) ≥ 1 and a p-value ≤ 0.05, indicating statistically significant differential expression. Hierarchical clustering was performed using the gplots package in R [49, 50]. All RNA extractions and sequencing were performed in biological triplicates.

## Supplementary Information

Below is the link to the electronic supplementary material.

Additional file 1. Figure S1. Growth and nybomycin production of S. explomaris 4N24 using minimal medium with different individual sugars.

## Data Availability

Data is provided within the manuscript or supplementary information files.
