# Supplementary material for: Metabolic engineering of Streptomyces explomaris for increased production of the reverse antibiotic nybomycin
Source: Microb Cell Fact. 2025 Oct 30;24:227. doi: 10.1186/s12934-025-02860-4 (PMC12573870; doi:10.1186/s12934-025-02860-4)
Supplement: Supplementary file 1 — Additional file 1. Figure S1. Growth and nybomycin production of S. explomaris 4N24 using minimal medium with different individual sugars. [file 12934_2025_2860_MOESM1_ESM.docx]

**Additional file 1 to**

**Metabolic engineering of *Streptomyces explomaris* for increased production of the reverse antibiotic nybomycin**

Submitted to *Microbial Cell Factories*

Wei Shu^1^, Julian Stegmüller^1^, Martha Rodriguez-Estevez^2^, Christian Rückert-Reed^3^, Jörn Kalinowski^3^, Oleksandr Gromyko^4,5^, Yurko Rebets^6^, Andriy Luzhetskyy^2^, and Christoph Wittmann^1^*

^1^ Institute of Systems Biotechnology, Saarland University, Saarbrücken, Germany

^2^ Pharmaceutical Biotechnology, Saarland University, Saarbrücken, Germany

^3^ CEBITEC, Bielefeld University, Germany

^4^ Department of Genetics and Biotechnology, Ivan Franko National University of Lviv, Ukraine

^5^ Microbial Culture Collection of Antibiotic Producers, Ivan Franko National University of Lviv, Ukraine

^6^ Explogen LLC, Lviv, Ukraine


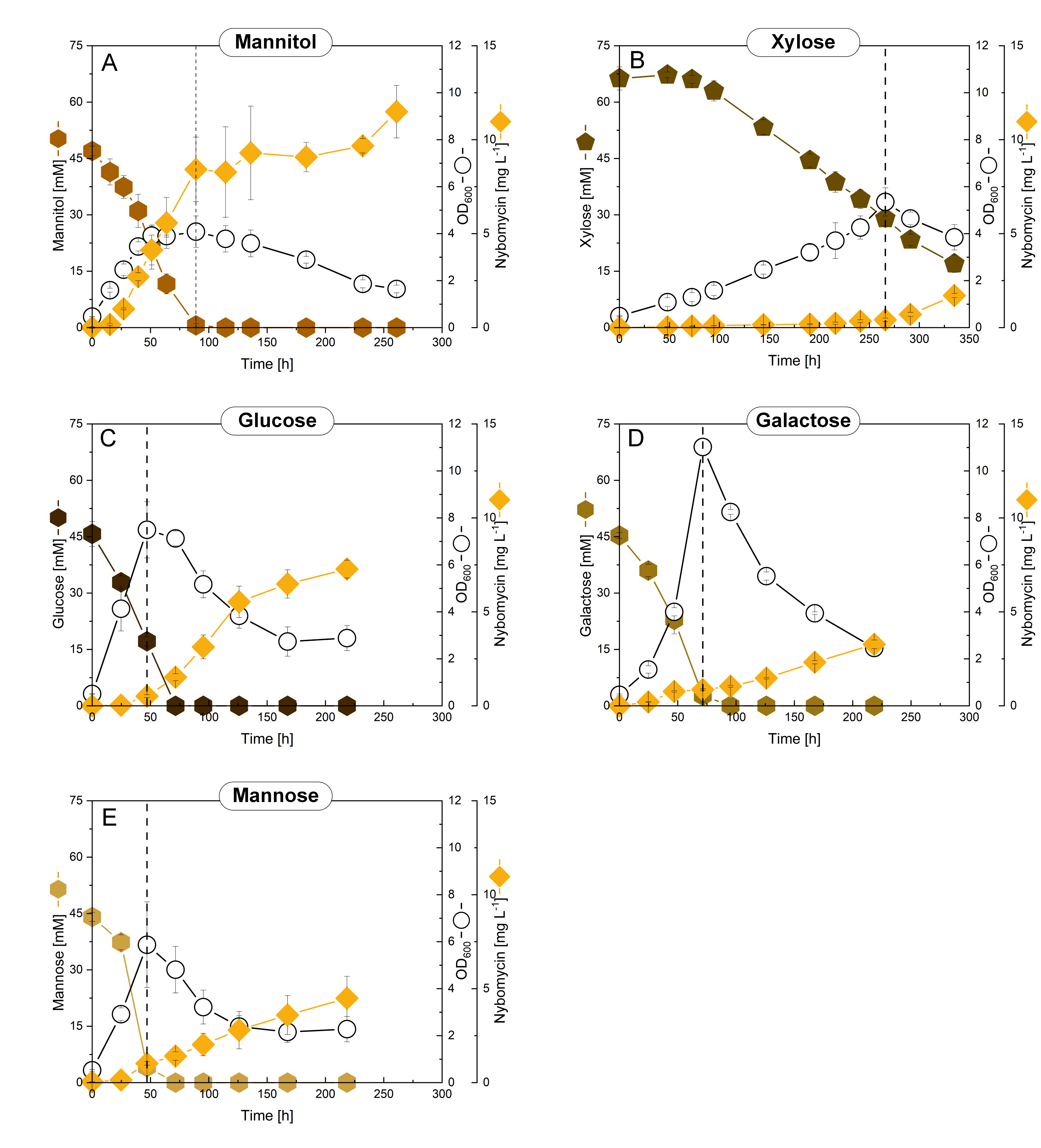


**Figure S1. Growth and nybomycin production of *S. explomaris* 4N24 using minimal medium with different individual sugars.**
